# The Metric System

**Published:** 1899-11

**Authors:** 


### THE METRIC SYSTEM.

If one will consult works on practice and materia medica of a score of years ago, he will probably find the more or less confident assertion in all of them, that the metric or decimal system of prescribing will soon supersede the then and now method. It is almost amusing to observe with what tenacity writers cling to this old garment of belief and continue to prophesy for it a modishness which it has never yet attained and probably never shall.

How many practicing physicians use the metric system? Despite its vaunted accuracy and convenience, very, very few think of employing it in preference to the familiar grains, drams, and ounces. A few with prepossessions established by "finishing" abroad, make feeble efforts to keep it up on their return, but like a good many things learnt abroad, it desquamates after the swelling of a "European education" has subsided.

The counter-prophecy may be hazarded that the decimal system will never take the place of the apothecary weight plan among American physicians. The latter is too firmly grafted upon the traditions of the Anglo-Saxon race. When the English give up their L. S. and D., and the merchants sell ginghams by the meter and kerosene by the litre, and lard by the kilo, we may look out for the era of "*nul, comma eins*." In the meanwhile we shall go on in the error of our way nurturing the belief that the sick will recover as quickly under the grain as the gram *régime*.

There is not, to our minds, any special reason for any change and so long as we do employ the apothecary weight in prescribing let us be consistent and employ it in our writings. What is so suggestive of offensive affectation as to see the metric system used by an American writer in an American medical journal. It not only disturbs the continuity and smoothness of reading by the pause for the mental arithmetic of reducing the unfamiliar to the familiar, but it does it so annoyingly that the reader has to repress a growing contempt for the writer. It savors too much, to turn to the expressive slang of the day, of "hot stuff"—an easy assumption of knowledge that is distinctly rasping.

Let us protect our own institutions. Good or bad they are ours, and so long as they are not wholly bad let loyalty triumph over "made in Germany" fads and innovations. W.

